# Surgery cancellations after entering the operating room

**DOI:** 10.1186/s40981-016-0066-1

**Published:** 2016-11-25

**Authors:** Yoko Hori, Ayami Nakayama, Atsuhiro Sakamoto

**Affiliations:** Department of Anesthesiology, Nippon Medical School, Sendagi 1-1-5, Bunkyo-ku, Tokyo, 113-8603 Japan

**Keywords:** Cancellation, General anesthesia, Surgery

## Abstract

**Background:**

Surgery cancellation results in unavailability of the operating room and loss time. We identified the frequency of and reasons for operation cancellations after patients entered the operating room and assessed the preventability of such cancellations.

**Findings:**

A retrospective chart review of all scheduled surgical procedures proposed under general anesthesia in a period spanning 2008 to 2016 was performed, and the reasons for cancellation were assessed.

A total of 30 surgery procedures were cancelled after the patient had entered the operation room and preparation for general anesthesia had been completed. Ten of 18 cases (55.6%) that were cancelled before general anesthesia induction could have been prevented, accounting for 36.7% of the overall cancellations. The majority of the cancellations after anesthesia were due to the patients’ health status.

**Conclusions:**

Improving the systems for checking patients’ medical problems and performing preoperative evaluations can reduce the number of cancellations after the patient has entered the operating room.

## Findings

### Background

Cancellations of surgery are costly and cause loss of hospital income. In addition, cancellations give a negative impression to patients. In particular, if a cancellation occurs after the patient has entered the operating room, it results in unnecessary costs and ineffective utilization of hospital resources and causes emotional distress to the patient. It is therefore necessary to reduce such cancellations as much as possible. Many studies have suggested methods for preventing cancellation of surgery due to patients’ medical problems, incomplete preoperative evaluations, system reasons, and other reasons [1, 2]. In this retrospective review of 30 cases with unexpected cancellation of surgery after the patient had entered the operating room, we analyze the indications for cancellations and offer suggestions for decreasing unexpected surgery cancellation.

### Methods

The authors performed a retrospective chart review of all scheduled surgical procedures under general anesthesia between December 1, 2008, and April 30, 2016, at the Nippon Medical School Hospital. We selected records of any surgery that was cancelled after the patient had already entered the operating room, before or after the induction of general anesthesia. Information about the patient and the reason for cancellation were obtained from the anesthesia record system, ORSYS® (Philips Electronics Japan, Tokyo), and the hospital’s electronic medical records. The ethics committee of the Nippon Medical School approved this study.

### Results

A total of 30 out of 51,933 surgical procedures scheduled between December 1, 2008, and April 30, 2016 were cancelled after the patient had entered the operation room and preparation for general anesthesia had been completed. The patient characteristics and American Society of Anesthesiologists physical status are shown in Table 1, and surgical specialties are shown in Table 2.

**Table 1:** Details of the patients’ characteristics of those who had their surgeries cancelled

Patient characteristics	Total (*n* = 30)
Mean age (years)	61.5 ± 22.1 (0.25–89)
Sex (male/female)	18/12
Operating room time (minutes)	44.3 ± 48.8 (20–217)
ASA physical status	
1	3 (10.0%)
2	18 (60.0%)
3	7 (23.3%)
4	2 (6.7%)
Surgery cancelled before anesthesia	18 (60.0%)
Surgery cancelled after anesthesia	12 (40.0%)

Data are means, mean ± SD, ranges (minimum–maximum), or number of patients.

**Table 2:** Operations cancelled by specialism

Operations cancelled by specialism	Total (*n* = 30)
Orthopedic surgery	11 (36.7%)
General surgery	5 (16.7%)
Vascular surgery	4 (13.3%)
Pulmonary surgery	3 (13.3%)
Ophthalmic surgery	3 (13.3%)
Surgery of Critical Care Medicine Center	2 (6.7%)
Gynecologic surgery	1 (3.3%)
Electric convulsive therapy	1 (3.3%)

Data show number of patients (and percentage)

The reasons for cancellation before induction of general anesthesia are shown in Table 3. The majority of cases were cancelled before anesthesia because of the patient’s health status. The possibility of surgery cancellation being preventable in these cases is shown also in Table 3. Two of four cases of atrial fibrillation were preventable, as this was overlooked on the preoperative 12-lead electrocardiograms. In all four shock cases, the surgeon decided in surgery that the patients’ hemodynamic state was too unstable due to their condition: intestinal necrosis, multiple injuries, excessive bleeding, or disseminated intravascular coagulation (DIC). An elective surgery had to be postponed as the patient had undergone percutaneous coronary intervention (PCI) 3 days earlier, which was not communicated to the surgeon beforehand. The cancellation of surgery in two hypoxemia and two fever cases was preventable, as the associated vital signs were observed on the day prior to the scheduled surgery in the general ward. A case of head injury just before entering the operation room received a prioritized head computed tomography (CT) scan prior to anesthesia. In another case, a pacemaker check could not be performed before surgery because of the lack of the appropriate medical equipment. Thus, in total, surgery cancellations could have been prevented in 10 (55.6%) of 18 cases before general anesthesia, accounting for 33.3% of overall cancellations.

**Table 3:** Cases surgery cancelled before anesthesia and cause of cancellation.

Surgery cancellation reason	Number	Preventable	Prediction is difficult
Patient health status			
Atrial fibrillation	4	2	2
Shock status	4	0	3
Hypertension	1	0	1
Cardiac arrest	1	0	1
After PCI	1	1	0
Hypoxemia	2	2	0
Fever	2	2	0
Head banging	1	1	0
System reason			
Necessity of pacemaker check	1	1	0
Patient issue			
Surgery rejection	1	1	0
Total	18	10 (55.6%)	8 (44.4%)

*PCI* percutaneous coronary intervention

Cases in which surgery was cancelled after anesthesia and the causes of cancellation are shown in Table 4. A change in the patient’s physical status after induction of general anesthesia, such as sudden drug-related anaphylactic shock, arrhythmias, and hypoxemia caused by atelectasis, is difficult to prevent. Regarding the outcomes after surgery cancellation, in cases of cancellation before anesthesia, 12 of 18 patients underwent the procedure at a later date. The condition of the three patients who were in shock died soon after cancellation, and one patient died within 24 days, which is a high mortality rate. In the cases of hypertension and cardiac arrest, the elective cataract surgery was not performed. In the cases of cancellation after anesthesia, 10 of 12 patients underwent the procedure at a later date after an appropriate solution was implemented. The remaining two cases received more conservative treatment.

**Table 4:** Cases surgery cancelled after anesthesia and cause of cancellation

Surgery cancellation reason	Total (*n* = 12)
Anaphylactic shock	3
Arrhythmias	
Arterial fibrillation	1
CAVB	1
TdP	1
Cardiac arrest	1
Hypoxemia	1
Anemia	1
Other	3

*CAVB* complete atrioventricular block, *TdP* Torsades de pointes

### Discussion

Our study highlights the role of insufficient evaluation of cases in which surgery is cancelled after the patient has entered the operating room. Several studies have shown the importance of preoperative anesthetic evaluation in the prevention of surgery cancellation [1–3]. Preoperative assessment by the anesthesiologist plays an important role in minimizing patient perioperative risk and preparation before surgery [4].

A Chinese study has reported that the rate of cancellations after patients entered the operating room was 0.21% [5]. A study of cardiac surgical case cancellations in Massachusetts indicated that 0.84% of such surgeries were cancelled after the patient had entered the cardiac surgical operating room [6]. In our study, the cancellation rate was less than 0.01%, which was relatively low. The probable reasons for this were as follows: there are very few day surgery cases in our hospital; all patients receiving general anesthesia should undergo a medical examination and evaluation by the anesthesiologist on the day before the operation [7, 8].

Our case review suggested that some of the cancellations could have been prevented or that the surgery could have been delayed before the patient entered the operating room and before anesthesia. In particular, the preoperative examinations planned by the attending surgeon should be checked, and vital signs at the ward should be confirmed by ward staff. A double-checking system could also be implemented, involving a nurse checking the essential preoperative examinations before the anesthesiologist does [9]. Requests for medical devices, such as a pacemaker, should be confirmed the day prior to surgery; not doing so constitutes a lack of communication.

Based upon our study, we propose the following preoperative evaluations. Patients’ vital signs (blood pressure, heart rate, body temperature, and oxygen saturation) should be measured accurately at their ward by the ward nurse and should be checked by the anesthesiologist. A structured preoperative assessment has been reported to improve operating room efficiency and reduce surgery cancellations [10]. A standardized preoperative evaluation checklist can also reduce the number of cancellations [11]. Required preoperative examinations, such as 12-lead electrocardiogram, laboratory analysis, and chest X-ray, should be confirmed before surgery and double-checked whenever possible. The patients’ medical history should be shared, and communication among the medical staff should be optimal.

Surgery cancellations after induction of general anesthesia are difficult to prevent, as the main reason for such cancellations is sudden and unexpected changes in the patient’s condition, such as anaphylactic shock or arrhythmia.

There were several limitations to this study. This was a retrospective, single-facility study, and the information was obtained only from medical records. No statistical analysis was performed as the number of cancellation cases was small.

### Conclusion

Improving the assessment of patients’ medical problems and preoperative evaluations can reduce the number of surgery cancellations after the patient has entered the operating room.

## Abbreviations

CT: Computed tomography
DIC: Disseminated intravascular coagulation
PCI: Percutaneous coronary intervention
